# Different Effects of Propofol and Isoflurane on Cochlear Blood Flow and Hearing Function in Guinea Pigs

**DOI:** 10.1371/journal.pone.0096861

**Published:** 2014-05-12

**Authors:** Ying Xiao, Jian Wen, Yanxia Bai, Na Duan, G X Jing

**Affiliations:** 1 Department of Anesthesiology, The First Affiliated Hospital of Medical College of Xi'an Jiaotong University, Xi'an, P.R.China; 2 Department of Otolaryngology, The First Affiliated Hospital of Medical College of Xi'an Jiaotong University, Xi'an, P.R.China; University of Salamanca- Institute for Neuroscience of Castille and Leon and Medical School, Spain

## Abstract

**Objectives:**

To investigate the effects of isoflurane and propofol on mean arterial pressure (MAP), cochlear blood flow (CoBF), distortion-product otoacoustic emission (DPOAE), and the ultrastructure of outer hair cells (OHCs) in guinea pig cochleae.

**Methods:**

Forty-eight male guinea pigs were randomly assigned to one of six treatment groups. Groups 1 to 3 were infused (i.v.) with a loading dose of propofol (5 mg/kg) for 5 min and three maintenance doses (10, 20, or 40 mg kg^−1^·h^−1^, respectively) for 115 min. Groups 4 to 6 were inhaled with isoflurane at concentrations of 1.15 vol%, 2.30 vol% or 3.45 vol% respectively for 120 min. CoBF and MAP were recorded prior to and at 5 min intervals during drug administration. DPOAE was measured before, immediately after, and 1 h after administration. Following the final DPOAE test, cochleae were examined using scanning electron microscopy.

**Results:**

Propofol treatment reduced MAP in a dose-dependent manner. CoBF and DPOAE showed increases at propofol maintenance doses of 10 and 20 mg kg^−1^·h^−1^. Inhalation of isoflurane at concentrations of 2.30 vol% and 3.45 vol% reduced MAP and CoBF. DPOAE amplitude increased following inhalation of 1.15 vol% isoflurane, but decreased following inhalations of 2.30 vol% and 3.45 vol%. Cochlear structure was changed following inhalation of either 2.30 vol% or 3.45 vol% isoflurane.

**Conclusions:**

Propofol could decrease MAP and increase both CoBF and DPOAE without affecting OHC structure. Inhalation of isoflurane at concentrations >2.30 vol% decreased CoBF and DPOAE, and produced injury to OHCs.

## Introduction

During middle ear surgery, it is important to maintain a relatively blood-free surgical field, and this can be achieved by decreasing systemic blood pressure to a level of controlled and stable hypotension[1]. Several pharmacological agents can be used for this purpose, and include vasodilators, alpha 2A adrenergic agonists, beta adrenergic antagonists, magnesium sulphate, and anesthetics[1]–[3]. Among those choices, the use of anesthetics to achieve controlled hypotension is convenient and welcomed by anesthesiologist[4], [5]. However, anesthetics must be used with caution, because hypotension may result in decreased cochlear blood flow (CoBF), leading to cochlear damage.

Normal CoBF is critically important for maintenance of endocochlear potential and endolymph production[6]. The main blood supply for the cochlea is the terminal spiral modiolar artery (SMA), which is a branch of the anterior inferior cerebellar artery (AICA). Several studies have suggested that CoBF has an autoregulation similar to that involved in brain blood flow [7], [8]. It has been shown that both isoflurane and desflurane impaired cerebral autoregulation at minimum alveolar concentrations (MAC) of 1.5, whereas propofol (200 µg·kg^−1^·min^−1^) was found to preserved it[9]. Ogawa[10] reported that sedations conducted using midazolam and propofol produced different effects on dynamic cerebral autoregulation, despite causing equivalent decreases in steady-state cerebral blood flow velocity. However, it is unclear whether a volatile anaesthetic (isoflurane) and a lipophilic i.v. anaesthetic (propofol) might have different effect on CoBF.

CoBF and cochlear structure/function can be assessed using laser Doppler flowmetry (LDF), distortion-product otoacoustic emission (DPOAE) tests, and scanning electron microscopy, respectively[11], [12]. Using these techniques, we systematically studies the effects of different doses of isoflurane and propofol on CoBF and OHCs structure/function. The current study provides important information for maintaining controlled hypotension during middle ear surgery.

## Materials and Methods

### Animals

Forty-eight male pigmented guinea pigs (500–600 g) with normal Preyer's reflex were obtained from the Experimental Animal Center of the Medical College of Xian Jiaotong University. Otomicroscopic examinations were performed to exclude the possibility of middle-ear pathology. Animals were housed in cages and maintained in environmentally controlled rooms with a 12-h light/dark cycle, and food and water available *ad libitum*. All experiments were approved by the Institutional Animal Care and Use Committee of Xian Jiaotong University. Efforts were made to minimize both animal suffering and the number of animals used in the study.

### Preparation and Surgery

The animals were anesthetized by intraperitoneal injection of barbital sodium (40 mg/kg), and then tracheotomized, paralyzed with vecuronium bromide, and artificially ventilated. Expiratory isoflurane and PCO_2_ values were continuously monitored by a gas analyzer (Vamos plus, Draeger, Germany). Body temperature was measured by a rectal probe that was coupled to a thermal sensor, and was maintained at 38.0±0.2°C with a heated blanket. The right carotid artery was cannulated with a polyethylene cannula (PE-20) to permit measurements of systemic blood pressure (BP) and sampling of arterial blood for gas analysis. Arterial BP was measured by a pressure transducer (PS260,Edwards Life Sciences,USA) and continuously recorded. A second microcatheter was inserted into the left jugular vein for administration of drugs.

### Laser Doppler Assessment of CoBF

CoBF in the right ear was continuously recorded before and during the 2 h period of drug administration, using Attanasio's method[11]_ENREF_26. The head of each animal was fixed in a moveable head holder. The right cochlea was exposed by a ventrolateral approach. The mucosa overlying the cochlea was gently removed, with care taken to keep the bone dry. CoBF was measured with a laser Doppler flowmeter (PeriFlux PF3, Perimed, Sweden,). The needle-shaped probe (1.6 mm OD) was placed on the lateral wall of the basal turn of the cochlea with a micromanipulator, and blood flow signals were recorded using a three-channel chart recorder. MAP and CoBF were collected every 5 min. The mean values of MAP and CoBF over the 3–5 min prior to drug infusion were used as baseline values. Changes in CoBF are expressed as percent changes from baseline.

### Distortion product otoacoustic emission (DPOAE)

An otomicroscope was used to examine the ears of the guinea pigs for external ear canal and middle ear obstructions, and the external auditory meatuses were cleaned. The tympanic membranes were punctured at the anteroinferior quadrant with a capillary needle (30 µm diameter) to balance pressures inside and outside the membrane[13]. DPOAE measurements were recorded using an otoacoustic emission analyzer (Intelligent Hearing System SmartOAE,Miami, FL, USA). DPOAE testing was performed in a double-shielded indoor room with ambient noise ≤30 dB SPL. An Etymotic 10B+ probe (Etymotic Research, Elk Grove Village, IL, USA) was inserted into the external ear canal. The bandwidth of the cubic DPOAE responses (2f1–f2) was set to a frequency range of 2.0–8.0 kHz, and 4 points were sampled using a 16-bit D/A converter. Frequencies were acquired with an f2:f1 ratio of 1.22. Primary tone intensities were set to L1 = 70 dB SPL, and L2 = 65 dB SPL. A valid DPOAE data point measurement with response amplitude 3 dB above background and a 3 dB signal-to-noise ratio (SNR) was considered to be positive. Distortion product-grams were acquired, and ≥2 assessments were obtained for both ears of each animal. Data obtained from the right ear are reported in the present paper.

### Scanning electron microscopy

Following the third DPOAE test, all animals were deep anesthetized with pentobarbital sodium prior to undergoing aortic cannulation and subsequent perfusion fixation with paraformaldehyde. Then, the bilateral auditory vesicle was removed, and the paries externus ductus cochlearis and stria vascularis were exorcized under a microscope. The excised tissues were fixed overnight in 3 vol% glutaraldehyde. The tissues were then dehydrated, dried, subjected to ion sputtering coating, and the OHCs were observed with a scanning electron microscope.

### Drug Treatment Procedure

A total of 48 animals were randomly assigned to one of six treatment groups (8 animals per group). Animals in groups 1–3 were infused (i.v.) with a 5 mg/kg loading dose of propofol (AstraZeneca, UK, Limited) for 5 min, followed by three different maintenance doses (10 mg kg^−1^·h^−1^, 20 mg·kg^−1^ h^−1^, or 40 mg·kg^−1^ h^−1^), for 115 min. Animals in groups 4–6 were inhaled with isoflurane (Baxter Pharmaceutical Products Inc, USA) at concentrations of 1.15 vol%, 2.30 vol%, and 3.45 vol%, respectively for periods of 120 min. Readings of CoBF and MAP were continuously monitored and recorded both prior to and at 5 min intervals during drug administration. DPOAE measurements were taken before, immediately after, and 1 h after drug administration. Following the final DPOAE test, cochlear structure was examined by scanning electron microscopy.

### Statistical Analysis

Data are expressed as means ± SD. Differences between and within treatment groups were measured by one- or two-way analysis of variance (ANOVA) for repeated-measures. When statistical significance was present, the post-hoc Student-Newman-Keuls test was used for multiple comparisons. All statistical analyses were performed using SPSS 13.0 software, and P-values <0.05 were considered statistically significant.

## Results

### Effects of propofol on MAP, CoBF, and DPOAE

**Figure 1:**
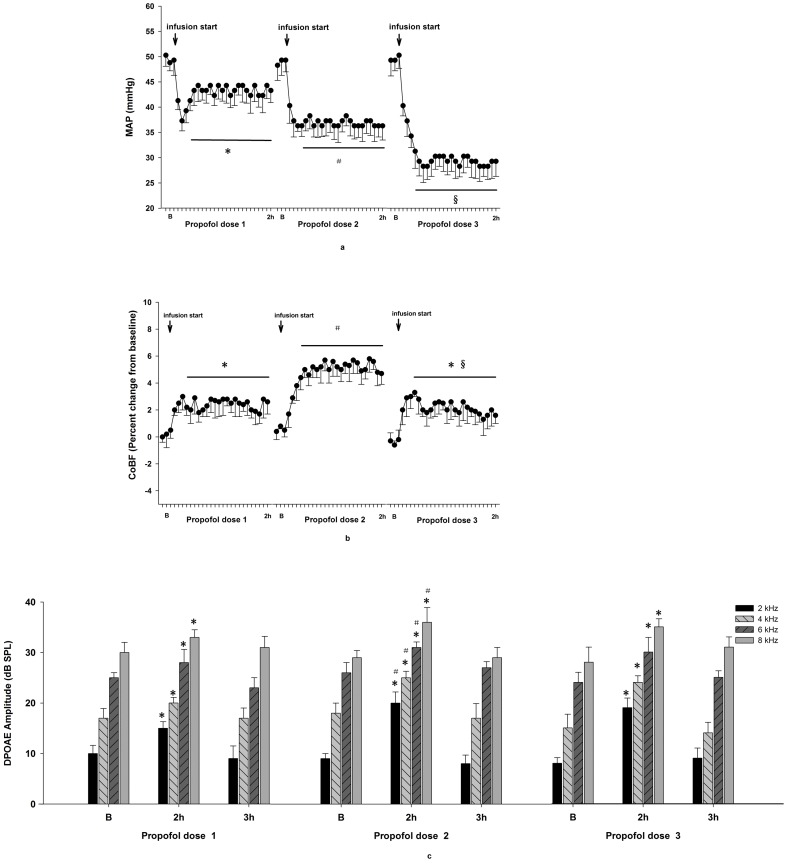
Effects of propofol on MAP, CoBF, and DPOAE. Following a loading dose of 5/kg, three propofol maintenance doses of 10 mg·kg^−1^ h^−1^ (propofol dose 1), 20 mg·kg^−1^·h^−1^ (propofol dose 2), and 40 mg kg^−1^ h^−1^ (propofol dose 3) were infused for 115 min. A, MAP values. * P<0.05 MAP 20 min after infusion vs B. # P<0.01 MAP 20 min after infusion vs B. § P<0.005 MAP 20 min after infusion vs B. B, CoBF values. * P<0.05 CoBF 20 min after infusion vs B. # P<0.01 CoBF 20 min after infusion vs B. § P<0.05 CoBF of propofol dose 3, 20 min after infusion vs CoBF of propofol dose 2, 20 min after infusion. C, DPOAE amplitudes. * P<0.05 DPOAE amplitude 2 h after infusion vs B. # P<0.05 DPOAE amplitude 2 h after infusion of propofol dose 2 vs DPOAE amplitude 2 h after infusion of propofol dose 1.

Compared with baseline values, the different doses of propofol caused decreases in MAP and increases in CoBF, and the changes became stabilized after a 20 min infusion time. The amount of decrease in MAP was dependent on the maintenance dose of propofol. CoBF at a maintenance dose of 20 mg·kg^−1^·h^−1^ was significant higher than that at 40 mg kg^−1^·h^−1^ (P = 0.0391, Figures 1a and 1b).

Compared with baseline values, DPOAE readings at 4 different frequencies (2, 4, 6, and 8 kHz) increased during 2 h infusions of three doses of propofol (P<0.05); however, readings recovered to baseline levels 1 h after the stop of infusion with every dose. DPOAE readings at 4 different frequencies (2, 4, 6, and 8 kHz) were higher at a propofol dose of 20 mg kg^−1^·h^−1^ than at a dose of 10 mg·kg^−1^ h^−1^ (P values  = 0.0003, 0.0003, 0.0313, and 0.0163, respectively), and were similar to readings at the same frequencies when using a 40 mg·kg^−1^·h^−1^ dose (P values  = 0.1854, 0.2753, 0.5368, and 0.4901, respectively, Figure 1c).

### Effects of isoflurane on MAP, CoBF, and DPOAE

**Figure 2:**
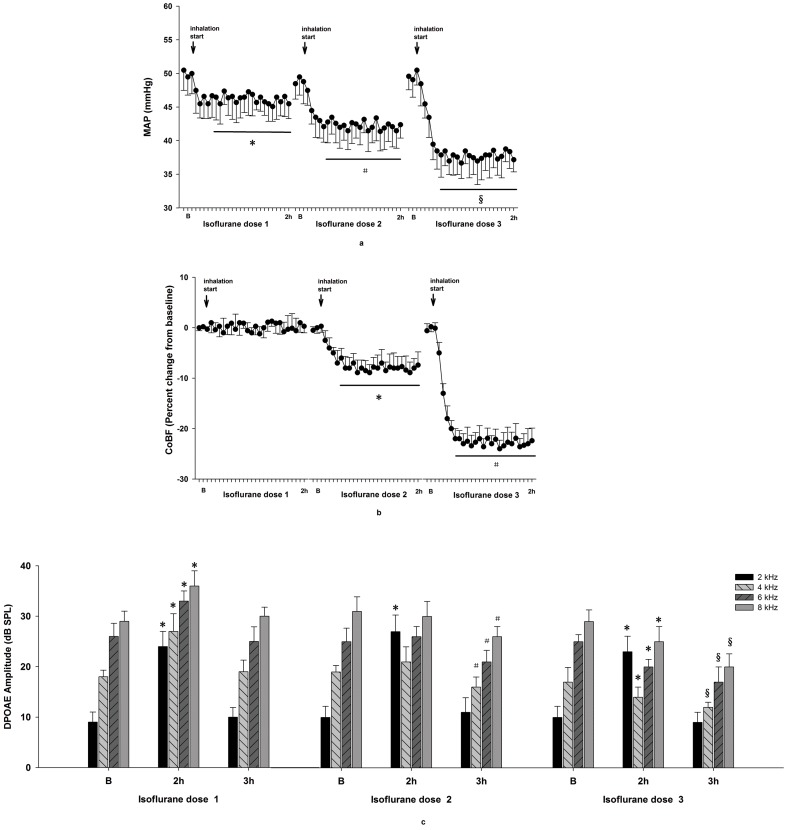
Effects of isoflurane on MAP, CoBF, and DPOAE. 1.15% (isoflurane dose 1), 2.30 vol% (isoflurane dose 2), and 3.45 vol% (isoflurane dose 3) were inhaled for 2 h. A, MAP values. * P<0.05 MAP 20 min after inhalation vs B. # P<0.01 MAP 20 min after inhalation vs B. § P<0.005 MAP 20 min after inhalation vs B. B, CoBF values. * P<0.01 CoBF 25 min after inhalation vs B. # P<0.005 CoBF 25 min after inhalation vs B. C, DPOAE amplitudes. * P<0.01 DPOAE amplitude 2 h after inhalation vs B. # P<0.01 DPOAE amplitude 1 h after the stop of isoflurane dose 2 vs B. § P<0.05 DPOAE amplitude 1 h after the stop of isoflurane dose 3 vs DPOAE amplitude 1 h after the stop of isoflurane dose 2.

Three different doses of isoflurane caused decreases in MAP, and the amounts of the decreases were dependent on the maintenance concentration administered. Additionally, the decreases in MAP stabilized after 25 min of isoflurane inhalation at levels significantly lower than those a baseline (P values  = 0.0021, 0.0009, and 0.0001, respectively). Isoflurane did not change CoBF, significantly decreased CoBF, and further decreased CoBF at levels of 1.15vol%, 2.30vol%, and 3.45vol%, respectively. Following inhalation of isoflurane for 25 min, CoBF in the 2.30vol% and 3.45vol% groups stabilized, but remained significantly lower than baseline values (P values  = 0.0018 and 0.0000, respectively, Figures 2a and 2b).

Compared with baseline values, DPOAE measurements at 4 different frequencies (2, 4, 6, and 8 kHz) increased during a 2 h inhalation of 1.15 vol% of isoflurane (P values  = 0.0000,0.0000, 0.0019, and 0.0014, respectively); However, the values returned to baseline 1 h after the stop of inhalation (P values  = 0.3807, 0.3661, 0.5792, and 0.1805, respectively). DPOAE measurements were significantly increased by 2.30 vol% isoflurane only at a frequency of 2 kHz (P = 0.0000), and there were no differences at 4, 6 and 8 kHz (P values  = 0.3063, 0.5727, and 0.8934, respectively). Compared with baseline DPOAE measurements returned to baseline values at 2 kHz (P = 0.3250), but decreased significantly at 4, 6, and 8 kHz (P values  = 0.0069, 0.0098, and 0.0008, respectively) 1 h after stopping inhalation. DPOAE was also significantly increased by 3.45 vol% isoflurane at 2 kHz (P = 0.0001), but measurements returned to baseline 1 h after the stop of inhalation (P = 0.4608). However, DPOAE measurements at 4, 6, and 8 kHz were significantly decreased by 3.45 vol% isoflurane (P values  = 0.0280, 0.0012, and 0.0262, respectively) compared with baseline after 2 h of inhalation and remained decreased at readings lower than in the 2.30 vol% group 1 h after the stop of inhalation (P values  = 0.0021, 0.0392, and 0.0000, respectively, Figure 1c).

### Observations of OHCs by scanning electron microscopy

**Figure 3:**
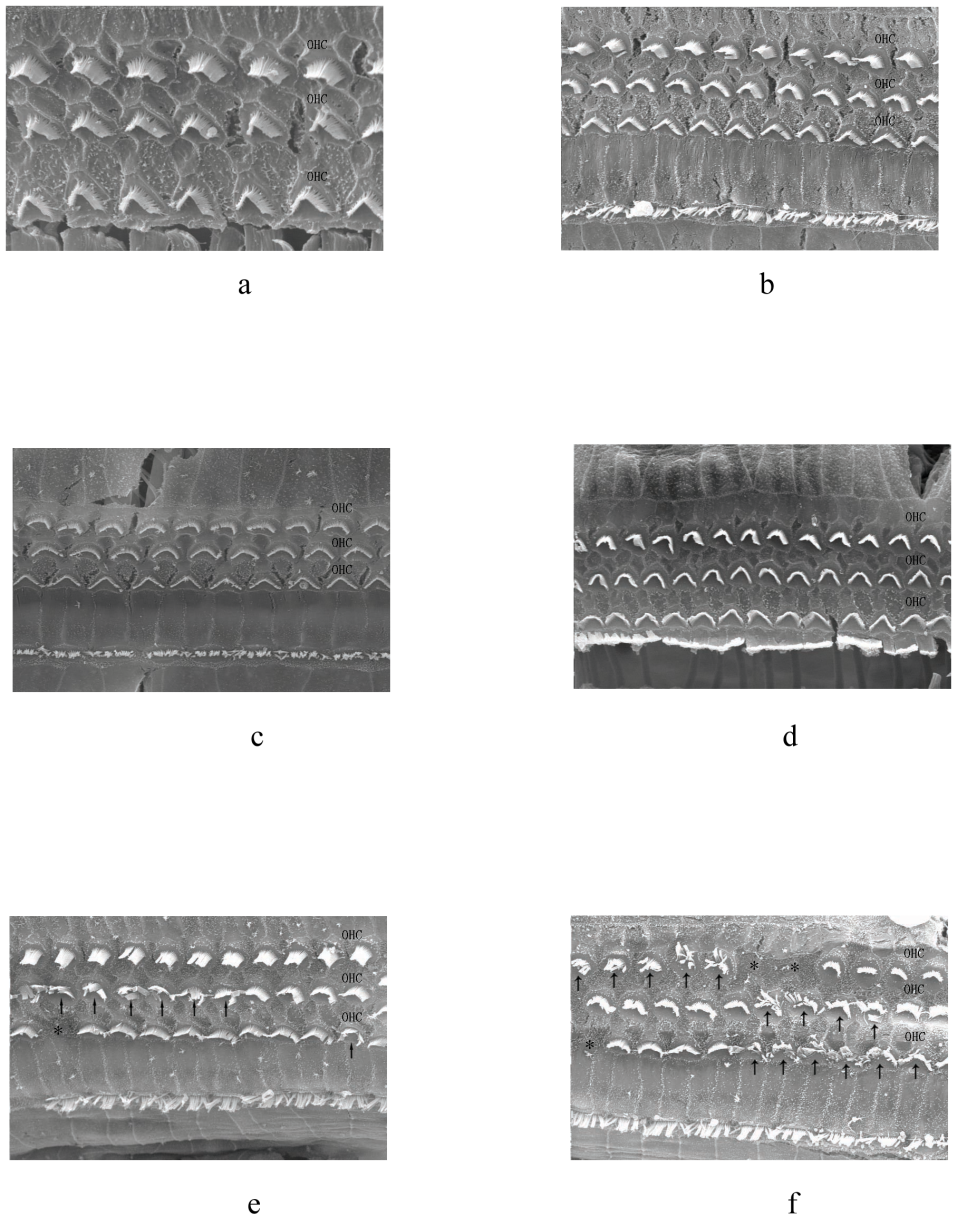
Scanning electron microscopy observations of OHCs. OHCs were well-arranged with normal ciliary structure at three doses of propofol (3a, b, c) and 1.15 vol% isoflurane (3d). Following inhalation of 2.30 vol% isoflurane, certain areas of OHCs were disordered and showed swelling and lodging cilia (3e)(marked by arrows). Following inhalation of 3.45 vol% isoflurane, numerous OHCs had disparate cilia and OHCs were absent in some regions (3f) (marked by asterisk).

Following administration of 3 different doses of propofol (Figures 3a, 3b, and 3c) or 1.15 vol% of isoflurane (Figure 3d), examination of OHCs by scanning electron microscopy showed normal cell arrangement and ciliary structure. However, treatment with 2.30 vol% isoflurane resulted in disordered OHCs arrangement with swelling and lodging cilia (Figure 3e). Treatment with 3.45 vol% isoflurane resulted in disparate cilia or the absence of cilia in certain regions (Figure 3f).

## Discussion

Anesthetics can be used to decrease systemic blood pressure to a level of controlled hypotension, which is very important for maintaining a relatively clear surgical field during middle ear surgery. In the present study, we found that propofol could decrease MAP and increase CoBF and DPOAE, without affecting OHCs structure. A 1.15 vol% isoflurane concentration could decrease MAP and increase DPOAE, without affecting CoBF or OHCs structure; however, higher concentrations of isoflurane decreased CoBF and DPOAE with injury to OHCs. These results suggest that isoflurane and propofol have different effects on CoBF, as well as OHCs structure and function.

While general anesthetics can affect systemic blood pressure, their effects on local organ perfusion depend on characteristics of organ specificity, drug specificity, and dose specificity. In the present study, we found that certain doses of propofol decreased MAP and increased CoBF, and these changes stabilized after 20 min of infusion. The decreases in MAP might be due to the vasodilatory effect of propofol on the microvascular system. It has been shown that propofol can dilate isolated arterioles via L-type calcium channels (LTCCs), resulting in hypotension[14]. However, increases in CoBF coincided with decreases in MAP, because propofol has vasodilatory effects and does not suppress autoregulation of CoBF which makes CoBF not affected by the decreasing of MAP. A previous study found that inner-ear blood flow remained relatively stable or autoregulated in guinea pigs at BP levels between 20 and 70 mmHg[7]. However, CoBF at the propofol maintenance dose of 20 mg kg^−1^ h^−1^ was significant higher than at a dose of 40 mg kg^−1^ h^−1^. This result might have occurred because the drop in MAP exceeded the working range of CoBF autoregulation, and overwhelmed potential improvement in CoBF by propofol. Different doses of isoflurane caused various decreases in MAP. Compared with the baseline values, 1.15 vol% of isoflurane did not alter CoBF, 2.30 vol% significantly decreased CoBF, and 3.45 vol% decreased CoBF even further. These effects might occur because higher concentrations of isoflurane may disrupt autoregulation of CoBF, thus making it easier for CoBF to be affected by fluctuations in blood pressure. In Preckel's study[15], controlled hypotension was maintained using sodium nitroprusside in patients anesthetized with propofol or isoflurane, and CoBF was autoregulated under propofol, but not under isoflurane during periods of decreasing BP. While single doses of propofol and isoflurane were utilized in Preckel's study, our current study utilized 3 different doses of two different anesthetics, and the results indicated that autoregulation of CoBF might be damaged by isoflurane at doses >2.30 vol%. These results suggested that propofol might have less of an effect on CoBF than isoflurane when used during middle ear surgery.

Distortion product otoacoustic emission (DPOAE) reflects the activity of cochlear OHCs, and hence provides a method for objectively assessing cochlear sensitivity. Analysis by DPOAE is objective, noninvasive, convenient, and can be used to detect early minor injuries resulting from noise, ototoxic drugs, ischemia, and hypoxia[12]. Additionally, DPOAE results are regulated by the brain stem auditory center. The medulla superior olivary nucleus can affect OHCs activity by synapses between the olivocochlear tract and OHCs, which usually suppress active mobility of OHCs[16]. Harel[17] found that under conditions of general anesthesia, DPOAE amplitudes were 30% higher than amplitudes in a conscious state, possibly because general anesthetics reduced inhibition of OHCs activity by the superior olivary nucleus, and the inhibitory effect decreased with increasing depth of anesthesia. Boyev[18] reported that a pentobarbital significantly increased DPOAE by diminishing medial olivocochlear reflex strength, whereas fentanyl and droperidol did not demonstrate such an effect. Other agents such as ketamine and nitrous oxide might affect DPOAE by raising or lowering tympanic pressure[19]. In the present study, the tympanic membrane was punctured at the anteroinferior quadrant with a capillary needle (30 µm diameter) to balance the inside and outside pressures and avoid the effect of general anesthetics on tympanic pressure. We found that DPOAE taken at all test frequencies (2, 4, 6, and 8 kHz) increased during 2 h of infusion with three different doses of propofol and 1.15 vol% isoflurane, but the measurements recovered to baseline levels 1 h after the stop of drug administration. Buki[20] found that DPOAE readings exhibited broadband increases caused by inhibition of the olivocochlear tract at all frequencies tested, which is consistent with the results of our current study. The return to baseline 1 h after the stop of anesthetics might be due to the washout of drugs which relieved inhibition of the superior olivary nucleus.

We found that DPOAE was significantly increased by inhalation of 2.30 vol% isoflurane only at the frequency of 2 kHz, and no differences were found at 4, 6, and 8 kHz. DPOAE was also significantly increased by inhalation of 3.45 vol% isoflurane at the frequency of 2 kHz, but was significantly decreased by 3.45 vol% of isoflurane at frequencies of 4, 6, and 8 kHz, and further decreases were found 1 h after the stop of inhalation. The reason for these findings may be that 2.30 vol% and 3.45 vol% isoflurane caused significant reductions in CoBF, resulting in cochlear damage. Previous studies revealed that inner ear ischemia induces decreases in DPOAE amplitude mainly at high frequencies[21], [22] which was consistent with our present research. Therefore, the effect of isoflurane on DPOAE reflected both decreased CoBF and inhibition of the olivocochlear tract.

Additionally, examinations by scanning electron microscopy showed that OHCs were well-arranged with normal ciliary structure at three different doses of propofol and 1.15 vol% isoflurane. However, treatment with 2.30 vol% isoflurane resulted in disordered OHCs arrangement, along with swollen and lodging cilia, and treatment with 3.45 vol% isoflurane resulted in disparate cilia and areas in which cilia were absent. These results suggested that higher concentrations of isoflurane could cause injury to OHCs, probably resulting from decreased CoBF and ischemia.

## Conclusions

When administered within a certain range of doses, propofol can increase both CoBF and DPOAE amplitude, without affecting OHCs structure. However, high concentrations of isoflurane (>2.30 vol%) can decrease CoBF and DPOAE, resulting in injury to OHCs. Our results suggest that isoflurane and propofol have different effects on cochlear structure and function when used to maintain controlled hypotension.

## References

[1] Ryu JH, Sohn IS, Do SH (2009) Controlled hypotension for middle ear surgery: a comparison between remifentanil and magnesium sulphate. Br J Anaesth 103: 490–495. 19687032 10.1093/bja/aep229

[2] Degoute CS, Ray MJ, Gueugniaud PY, Dubreuil C (2003) Remifentanil induces consistent and sustained controlled hypotension in children during middle ear surgery. Can J Anaesth 50: 270–276. 12620951 10.1007/BF03017797

[3] Firat Y, Kizilay A, Akarcay M, Yucel A, But K, et al (2007) The effect of dexmedetomidine on middle ear pressure. Otolaryngol Head Neck Surg 137: 218–223. 17666244 10.1016/j.otohns.2007.03.005

[4] Kol IO, Kaygusuz K, Yildirim A, Dogan M, Gursoy S, et al (2009) Controlled hypotension with desflurane combined with esmolol or dexmedetomidine during tympanoplasty in adults: A double-blind, randomized, controlled trial. Curr Ther Res Clin Exp 70: 197–208. 24683230 10.1016/j.curtheres.2009.06.001 PMC3967361

[5] Powell JT, Hinchliffe RJ, Thompson MM, Sweeting MJ, Ashleigh R, et al (2014) Observations from the IMPROVE trial concerning the clinical care of patients with ruptured abdominal aortic aneurysm. Br J Surg 101: 216–224 discussion 224. 24469620 10.1002/bjs.9410 PMC4164272

[6] Shi X (2011) Physiopathology of the cochlear microcirculation. Hear Res 282: 10–24. 21875658 10.1016/j.heares.2011.08.006 PMC3608480

[7] Brown JN, Nuttall AL (1994) Autoregulation of cochlear blood flow in guinea pigs. Am J Physiol 266: H458–467. 8141346 10.1152/ajpheart.1994.266.2.H458

[8] Kawakami M, Makimoto K, Fukuse S, Takahashi H (1991) Autoregulation of cochlear blood flow. A comparison of cerebral blood flow with muscular blood flow. Eur Arch Otorhinolaryngol 248: 471–474. 1768409 10.1007/BF00627636

[9] Strebel S, Lam AM, Matta B, Mayberg TS, Aaslid R, et al (1995) Dynamic and static cerebral autoregulation during isoflurane, desflurane, and propofol anesthesia. Anesthesiology 83: 66–76. 7605020 10.1097/00000542-199507000-00008

[10] Ogawa Y, Iwasaki K, Aoki K, Gokan D, Hirose N, et al (2010) The different effects of midazolam and propofol sedation on dynamic cerebral autoregulation. Anesth Analg 111: 1279–1284. 20881283 10.1213/ANE.0b013e3181f42fc0

[11] Attanasio G, Buongiorno G, Piccoli F, Mafera B, Cordier A, et al (2001) Laser Doppler measurement of cochlear blood flow changes during conditioning noise exposure. Acta Otolaryngol 121: 465–469. 11508505

[12] Janssen T, Niedermeyer HP, Arnold W (2006) Diagnostics of the cochlear amplifier by means of distortion product otoacoustic emissions. ORL J Otorhinolaryngol Relat Spec 68: 334–339. 17065826 10.1159/000095275

[13] LeBourgeois HW 3rd, Anand VK, McAuley JR, Dickman JD, Malphurs O Jr (2000) Effect of tympanic perforations on the detection of distortion-product otoacoustic emissions. Ear Nose Throat J 79: : 610–612, 614–616, 618. 10969471

[14] Lawton BK, Brown NJ, Reilly CS, Brookes ZL (2012) Role of L-type calcium channels in altered microvascular responses to propofol in hypertension. Br J Anaesth 108: 929–935. 22511481 10.1093/bja/aes069

[15] Preckel MP, Ferber-Viart C, Leftheriotis G, Dubreuil C, Duclaux R, et al (1998) Autoregulation of human inner ear blood flow during middle ear surgery with propofol or isoflurane anesthesia during controlled hypotension. Anesth Analg 87: 1002–1008. 9806672 10.1097/00000539-199811000-00004

[16] Di Girolamo S, Napolitano B, Alessandrini M, Bruno E (2007) Experimental and clinical aspects of the efferent auditory system. Acta Neurochir Suppl 97: 419–424. 10.1007/978-3-211-33081-4_47 17691330

[17] Harel N, Kakigi A, Hirakawa H, Mount RJ, Harrison RV (1997) The effects of anesthesia on otoacoustic emissions. Hear Res 110: 25–33. 9282886 10.1016/s0378-5955(97)00061-0

[18] Boyev KP, Liberman MC, Brown MC (2002) Effects of anesthesia on efferent-mediated adaptation of the DPOAE. J Assoc Res Otolaryngol 3: 362–373. 12382109 10.1007/s101620020044 PMC3202411

[19] Hatzopoulos S, Petruccelli J, Laurell G, Finesso M, Martini A (2002) Evaluation of anesthesia effects in a rat animal model using otoacoustic emission protocols. Hear Res 170: 12–21. 12208537 10.1016/s0378-5955(02)00448-3

[20] Buki B, Wit HP, Avan P (2000) Olivocochlear efferent vs. middle-ear contributions to the alteration of otoacoustic emissions by contralateral noise. Brain Res 852: 140–150. 10661505 10.1016/s0006-8993(99)02227-1

[21] Hamed SA, El-Attar AM (2010) Cochlear dysfunction in hyperuricemia: otoacoustic emission analysis. Am J Otolaryngol 31: 154–161. 20015733 10.1016/j.amjoto.2008.12.002

[22] Morawski K, Telischi FF, Merchant F, Abiy LW, Lisowska G, et al (2003) Role of mannitol in reducing postischemic changes in distortion-product otoacoustic emissions (DPOAEs): a rabbit model. Laryngoscope 113: 1615–1622. 12972944 10.1097/00005537-200309000-00039 PMC1769330

